# Receptor-Associated Protein (RAP) Plays a Central Role in Modulating Aβ Deposition in APP/PS1 Transgenic Mice

**DOI:** 10.1371/journal.pone.0003159

**Published:** 2008-09-08

**Authors:** Guilian Xu, Celeste Karch, Ning Li, Nianwei Lin, David Fromholt, Victoria Gonzales, David R. Borchelt

**Affiliations:** 1 Department of Neuroscience, McKnight Brain Institute, Santa Fe Health Alzheimer's Disease Research Center, University of Florida, Gainesville, Florida, United States of America; 2 Interdisciplinary Graduate Program, University of Florida, Gainesville, Florida, United States of America; 3 Department of Pathology, Johns Hopkins University School of Medicine, Baltimore, Maryland, United States of America; University of Birmingham, United Kingdom

## Abstract

**Background:**

Receptor associated protein (RAP) functions in the endoplasmic reticulum (ER) to assist in the maturation of several membrane receptor proteins, including low density lipoprotein receptor-related protein (LRP) and lipoprotein receptor 11 (SorLA/LR11). Previous studies in cell and mouse model systems have demonstrated that these proteins play roles in the metabolism of the amyloid precursor protein (APP), including processes involved in the generation, catabolism and deposition of β-amyloid (Aβ) peptides.

**Methodology/Principal Findings:**

Mice transgenic for mutant APPswe and mutant presenilin 1 (PS1dE9) were mated to mice with homozygous deletion of RAP. Unexpectedly, mice that were homozygous null for RAP and transgenic for APPswe/PS1dE9 showed high post-natal mortality, necessitating a shift in focus to examine the levels of amyloid deposition in APPswe/PS1dE9 that were hemizygous null for RAP. Immunoblot analysis confirmed 50% reductions in the levels of RAP with modest reductions in the levels of proteins dependent upon RAP for maturation [LRP trend towards a 20% reduction ; SorLA/LR11 statistically significant 15% reduction (p<0.05)]. Changes in the levels of these proteins in the brains of [APPswe/PS1dE9](+/−)/RAP(+/−) mice correlated with 30–40% increases in amyloid deposition by 9 months of age.

**Conclusions/Significance:**

Partial reductions in the ER chaperone RAP enhance amyloid deposition in the APPswe/PS1dE9 model of Alzheimer amyloidosis. Partial reductions in RAP also affect the maturation of LRP and SorLA/LR11, which are each involved in several different aspects of APP processing and Aβ catabolism. Together, these findings suggest a central role for RAP in Alzheimer amyloidogenesis.

## Introduction

The genetic alterations in APP and PS1 (and PS2) that cause early onset familial Alzheimer's disease (AD) are well characterized, as are the consequences that disease-linked mutations in these proteins have on the endoproteolytic processing of APP (for review see [1]). However, the cause of sporadic AD is less well defined. The major genetic risk factor for sporadic AD is the presence of the E4 allele of the apolipoprotein E (Apo E) gene [2]. Polymorphisms in the LRP and SorLA/LR11 genes have also been associated with increased risk for Alzheimer's disease [3], [4]. However, there are studies that refute the LRP finding [5]–[8].

LRP, a member of the low density lipoprotein (LDL) receptor family, is a large, multifunctional endocytic receptor, highly expressed in hepatocytes, fibroblasts, activated astrocytes, and neurons (reviewed by Willnow [9]). Mature LRP is composed of two subunits, 515 kDa (α-chain) and 85 kDa (β-chain), which are produced by proteolytic cleavage from a single polypeptide precursor of 600 kDa in the trans-Golgi network [10]. Maturation of LRP to the trans-Golgi network is partially dependent upon the presence of LRP-receptor-associated protein (RAP), which binds to LRP at multiple sites to block the receptor's ability to interact with its ligands [11]. Premature binding of LRP ligands to the receptor interferes with maturation of LRP, and similar receptors, and with translocation to the plasma membrane (reviewed by Willnow [12]).

LRP is a potentially important etiological agent for AD because three proteins clearly involved in AD - APP, APOE, and α2-macroglobulin (α2M), are ligands of LRP [for review see [13]]. Importantly, each of these proteins plays significant roles in the production and metabolism of Aβ peptide, the principal component of amyloid plaques that characterize AD. LRP has been found to bind to the amyloid precursor protein (APP) in a manner that alters its trafficking and processing [14]–[18]. LRP-deficient cells secrete less Aβ and restoring LRP function substantially increases Aβ production [15]. One study has reported reduced levels of LRP in the brains of AD patients [19], but this finding has not been confirmed by others [20]. LRP also appears to mediate the clearance of Aβ that is bound to α2M or ApoE[19], [21]–[23]. It is, therefore, important to examine the role of LRP in the pathogenesis of Alzheimer-type amyloidosis in transgenic mouse models of this pathology. Unfortunately, the large size of the LRP gene (>10 kb) makes production of transgenic animals that express the whole protein difficult by standard cDNA approaches. Zerbinatti et al. [24] reported that overexpression of an LRP minireceptor in the PDAPP mouse model of AD, resulted in increased levels of soluble Aβ but did not impact amyloid burden. Similarly, it is not possible to examine adult animals that lack LRP because targeted deletion of the LRP gene in mice leads to death of the embryo at day 13.5 [25]. However, the levels of functional LRP in the brain are regulated by RAP. Unlike LRP, mice deficient in RAP are viable and have normal lifespans because the maturation of LRP is only partially dependent upon RAP; neurons of RAP KO mice show 75% reductions in the levels of endoproteolytically processed, mature, LRP [26]. It is noteworthy that expression of PS1 variants M146L or L286V, which are linked to familial AD, also cause 40% reductions in the levels of mature LRP in brain [27].

In the present study, we have utilized RAP KO mice crossed with mice that co-express mutant APP (APPswe) and PS1 (PS1dE9) to examine how loss of RAP influences amyloid deposition. This work, in part, is a repetition of a previous effort by van Uden and colleagues in which RAP knockout mice were crossed to the PDAPP model of AD amyloidosis [28]. However, the present study differs from this previous work in a few important ways, providing new insights into the mechanisms involved. First, we determine the effects of partial loss of RAP on amyloid deposition by examining mice with heterozygous deletion of RAP. Second, we use mice that co-express mutant presenilin, which has been shown in other studies to reduce the maturation of LRP [27]. Third, we study mice that express the 695 isoform of APP, which lacks a domain critical for interaction with LRP. Also, we noted that methods described in the van Uden study indicated that mice heterozygous for the APP transgene and heterozygous for RAP [APP (+/−)/RAP(+/−)] were intercrossed to produce the animals ultimately analyzed: APP transgene positive and homozygous wild-type for RAP or homozygous null for RAP. A possible flaw in this strategy is that some offspring could be homozygous for the APP transgene (i.e. have two transgene alleles instead of one). If transgene homozygous animals were, by chance, over-represented in one of the RAP genotypes examined, then there could be error in determining the contribution of RAP to amyloid load. Thus, we repeated the study, using our APPswe/PS1dE9 model of AD and RAP KO mice. For reasons that are not clear, mice hemizygous for the APPswe/PS1dE9 transgenes and homozygous null for RAP died at high frequency just after birth. We therefore studied mice that were heterozygous null for RAP and heterozygous for the APPswe/PS1dE9 transgenes. The partial reduction in RAP led to very modest reductions in the levels of mature LRP and SorLA/LR11 (∼15–20% reductions of both) with predictable 50% reductions in the levels of RAP. The changes in the levels of these proteins correlated with 30–40% increases in amyloid burden (assessed at 9 months of age). Our findings indicate that RAP plays a focal role in the biology of amyloid deposition either by regulating the maturation of proteins that modulate APP processing and Aβ metabolism, or by directly modulating other yet to be defined aspects of amyloidogenesis.

## Methods

### Transgenic mice

The AD mouse model used in this study (APPswe/PS1dE9-Line 85) co-expresses a chimeric mouse/human APP695 harboring the Swedish K670M/N671L mutations (Mo/HuAPPswe) and human PS1with the exon-9 deletion mutation (PS1dE9). This model was generated by co-injection of MoPrP.Xho expression plasmids for each gene; the two transgenes co-integrated and segregate as a single locus [29].

Mice with targeted-deletions of RAP (Strain B6, 129S-Lrpap1^tm1Her^) were purchased from the Jackson Laboratories (stock # 002987; Bar Harbor, ME). These RAP knockout mice [RAP(−/−)], which were congenic in the C57BL/6J strain, were crossed to APPswe/PS1dE9 mice (Line 85), which were F2 hybrids of C57BL/6J and C3H/HeJ. Progeny that were [APPswe/PS1dE9](+/−)/RAP(+/−) were backcrossed to the congenic RAP−/− mice to hasten the production of mice that were APPswe/PS1dE9 transgene positive and RAP null.

### Tissue preparation, histology and amyloid burden measurements

All procedures involving animals were approved by the Johns Hopkins Institutional Animal Care and Use Committee. At the specified age, animals were euthanized by overdose with ethyl ether before the brains were removed and bisected sagittally. One hemibrain was frozen on dry ice and stored at −80°C for biochemical studies. The other hemibrain was immersion-fixed in 4% paraformaldehyde in phosphate-buffered saline (PBS pH 7.4). Later, the fixed tissues were embedded in paraffin for silver staining according to the Bielschowsky method [30] modified from the Hirano method (detailed description in [31]).

Estimation of amyloid plaque loads was performed by counting amyloid plaques in 6 sagittal sections through the hippocampus of 7 male [APPswe/PS1dE9](+/−)/RAP(+/−) mice and the 6 parental APPswe/PS1dE9-Line 85 male mice that were used in the initial cross to RAP(−/−) females. Images of hippocampus were captured by digital photography. The hippocampus was defined and the amyloid deposits contained within were counted separately by two people blinded to the genotype. The number of plaques in each section were summed and then averaged for each animal. Statistical analyses (2-tailed t-Test) were performed using the average number of deposits in hippocampus for each animal as a single data point.

### Detection of high-molecular-weight aggregates of Aβ

Amyloid burden was estimated biochemically by filter assay as previously described [32]. Briefly, mouse hemi-forebrains (cerebellum and brain stem removed) were homogenized by probe sonication in 10 volumes of Tris-HCl buffered saline (20 mM Tris-HCl, pH 8.0, 150 mM NaCl) with protease inhibitor. Six parental Line 85 mice (all male) and 7 [APPswe/PS1dE9](+/−)/RAP(+/−) mice (F1 and F2 offspring – all male) were used in this study. Homogenates were centrifuged at 800×g for 5 min in a microcentrifuge. The supernatant was adjusted to a final SDS concentration of 1% and then passed through cellulose acetate membranes, 0.2-µm pore size (OE66, Schleicher & Schuell, Keene, NH), using a 96-well dot-blot apparatus under vacuum. Proteins trapped by the filter were detected by immunostaining following protocols used in immunoblotting with a rabbit polyclonal antibody from Zymed, CA (Cat. #71–5800). Enhanced chemiluminescence (ECL) signal was digitally captured with LAS-3000 imaging system, and the intensity of the dots was quantified using Multi Gauge software (Fujifilm, Japan). Two-tailed student t-test with equal variance was used to estimate the difference between two groups.

### Western blot analysis

Levels of RAP, LRP, SorLA/LR11 and APP were assessed by western blot, using standard methods, as previously described [33]. Briefly, brains were homogenized as described above and then centrifuged at 800×g for 5 minutes. Portions of the supernatant, containing 100 µg or 50 µg of total protein, were separated by SDS-PAGE and transferred to nitrocellulose membranes. Blots were incubated with rabbit polyclonal antibody 377 αLRP (1∶1000, gift of Dr. J. Herz, University of Texas Southwestern Medical Center, Dallas, Texas, USA), rabbit polyclonal antibody 4109 αRAP (1∶1000, also a gift of Dr. J. Herz), SorLA/LR11antibody (1∶1000; monoclonal, BD Biosciences, San Jose, CA) or 6E10 Aβ (1∶5000, monoclonal, Signet Laboratories, Dedham, MA, USA). Proteins bound to antibodies were revealed by incubation with HRP conjugated secondary antibodies (KPL, Gaithersburg, Maryland) and chemiluminescence. The signal was captured and quantified using the LAS-3000 imaging system as described above. The statistical methods of analysis are described in the Figure Legends.

## Results

RAP(−/−) mice, first described by Willinow et al. [26], were obtained from the Jackson laboratories as congenic on the C57BL/6J background. Animals were mated to APPswe/PS1dE9- Line 85 mice (B6C3/F2) background to produce an F1 generation of mice that were [APP/PS1](+/−)/RAP(+/−), which we then backcrossed to RAP(−/−) mice in an attempt to produce mice that were [APP/PS1](+/−)/RAP(−/−). However, we unexpectedly failed to obtain weaning age mice that were [APP/PS1](+/−)/RAP(−/−) (Table 1). The mating scheme used was expected to yield litters in which 50% of the offspring were RAP(−/−), with roughly half of these offspring also harboring the APPswe/PS1dE9 transgene. Similarly 50% of the offspring should be RAP (+/−), with half of these offspring also harboring the APPswe/PS1dE9 transgene. However, weaning age offspring of the [APPswe/PS1dE9](+/−)/RAP(−/−) genotype were present at far lower percentages than expected; only 2 animals of this genotype reached weaning age to be identified and both of these died (of unknown causes) before reaching 3 months of age. These results suggested that some type of interaction between an activity of APPswe or PS1dE9 and the absence of RAP leads to diminished survival of these animals. Presently, we don't understand the basis for this outcome.

**Table 1:** Genotypes of offsprings between crosses of APPswe/PS1dE9(+)/RAP(+/−) mice and RAP (−/−) mice.

Genotype	Expected Frequency	Number of Offspring	Observed Frequency
APPswe/PS1dE9(+)/RAP(+/−)	25%	23	41.0%
APPswe/PS1dE9(+)/RAP(−/−)	25%	2*	3.5%
RAP(+/−)	25%	21	37.5%
RAP(−/−)	25%	10	17.8%

* Both animals died before 3 months of age.

### Partial reduction in RAP levels increases amyloid burden

**Figure 1:**
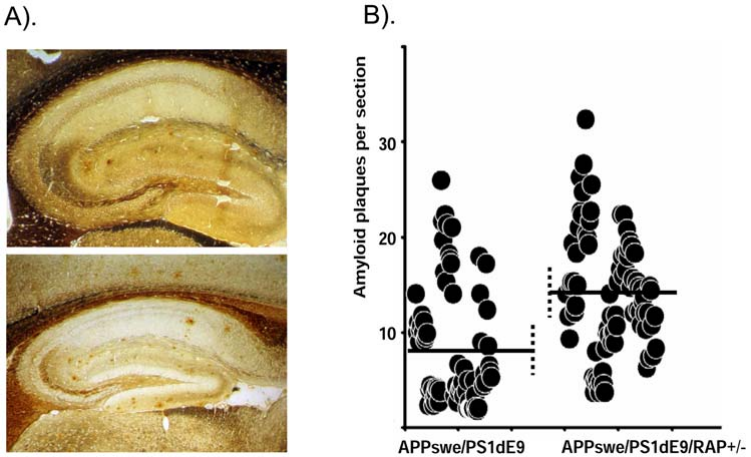
Partially reducing RAP increases Aβ deposition in the brains of APPswe/PS1dE9 mice. A). Silver staining reveals increased amyloid deposition in the brains of [APPswe/PS1dE9](+/−)/RAP(+/−) mice as compared to the parental APPswe/PS1dE9 line 85 mice. B). Plot of the results from counting amyloid plaques following procedures described in Methods. Each dot indicates the number of amyloid plaques on a section. Six sections per animal were counted by two independent assessors that were blind to the genotype of the animals. Each section was counted 3 times by each assessor and averaged. Statistical analyses was conducted on the mean number of deposits for each animal [n = 6 for APPswe/PS1dE9 mice and n = 7 for the [APPswe/PS1dE9](+/−)/RAP(+/−) mice]. The statistical difference in amyloid burden between animals of the 2 genotypes was estimated by 2-tailed t-test with equal variance (p<0.05).

**Figure 2:**
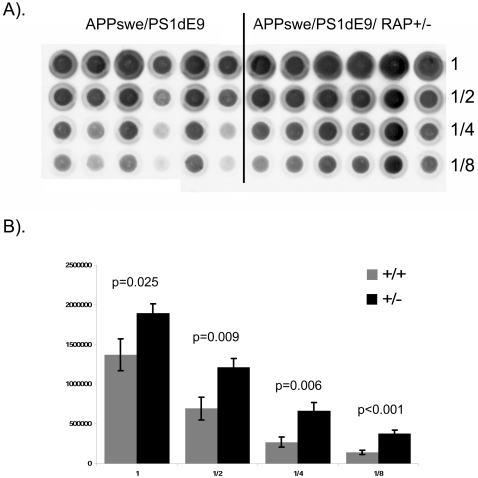
Filter assay of amyloid burden in [APPswe/PS1dE9](+/−)/RAP(+/−) mice. A). Filter trap assays of tissue homogenates from 6 parental line 85 mice and 6 offspring that were APPswe/PS1dE9 positive and hemizygous for RAP was performed as described in Methods. Each column contains a serial dilution of the sample. 300 µg total protein was loaded in the first well, followed by serial 2-fold dilutions. The [APPswe/PS1dE9](+/−)/RAP(+/−) mice have about twice the amount of high molecular weight Aβ burden as the parental line 85 mice at the same age (9 months). B). Quantification of signal intensity was measured as described in Methods across the entire range of dilutions for each group of mice. The amount of high molecular weight “aggregated” Aβ was found to be significantly higher in [APPswe/PS1dE9](+/−)/RAP(+/−) mice at 9 months as compared to the parental APPswe/PS1dE9 animals of the same age (p values noted on graph).

Because we were unable to produce [APPswe/PS1dE9](+/−)/RAP(−/−) mice, we chose to compare the parental lines of APPswe/PS1dE9 animals, which were wild-type with respect to RAP, to F1 and F2 [APPswe/PS1dE9](+/−)/RAP(+/−) mice. By multiple measures, we found that partial loss of RAP was associated with increased levels of amyloid deposition. Histologically, we found that partial deletion of RAP increased the number of amyloid deposits 1.5 fold in 9 month old mice (Figs. 1A and B). The average number of silver stained plaques in the hippocampus of [APPswe/PS1dE9](+/−)/RAP(+/−) mice was 13.5 as compared to 8.1 in the parental line 85 mice. Statistical analyses of the data estimated the difference in amyloid burden between the two genotypes has a low probability of resulting from random chance (<0.05). To further quantify the levels of aggregated β-amyloid in these mice, we used a filter-trap assay [32] to demonstrate that the brains of mice expressing APPswe/PS1dE9 with a partial loss of RAP contained about twice as much high-molecular-weight Aβ as controls (Fig. 2). Homogenates of forebrain were serially diluted (2-fold) and filtered through 0.2 µm cellulose acetate membrane, then immunoblotted as described in Methods. We determined that the forebrain of [APPswe/PS1dE9](+/−)/RAP(+/−) mice, as compared to [APPswe/PS1dE9](+/−)/RAP(+/+) mice, contained about twice as much Aβ immunoreactivity that was retained by the filter (p<0.025). Together, these findings provide evidence for higher levels of amyloid in [APPswe/PS1dE9](+/−)/RAP(+/−) mice.

### Partial reduction in RAP lowers the levels of SorLA/LR11 and LRP

**Figure 3:**
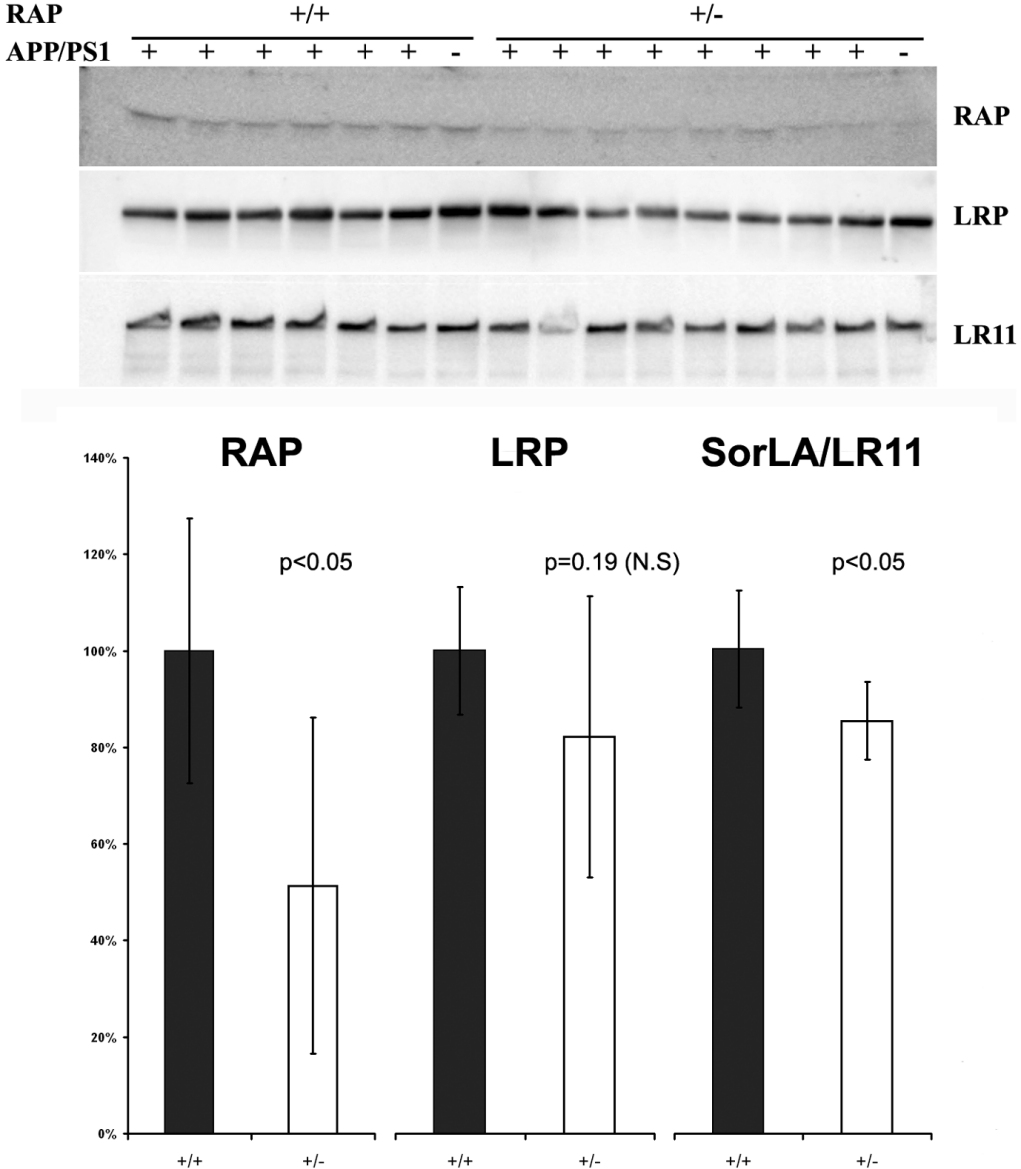
Analysis of RAP, LRP and SorLA/LR11 levels. A). Immunoblots were probed with polyclonal antibody 4109 to RAP (1∶1000), 377 to LRP (1∶1000) and monoclonal antibody anti-LR11 (1∶1000; BD Biosciences, San Jose, CA). Protein concentration was determined by BCA. Each lane contains 100 µg total protein. Genotypes of the mice are marked on the figure. B). Quantification of the intensity of the bands in panel A, using a Fuji LAS-3000 imaging device and software provided by the manufacturer. Statistical comparisons of each protein in the two genotypes of interest ([APPswe/PS1dE9](+/−)/RAP(+/+) and [APPswe/PS1dE9](+/−)/RAP(+/−) were conducted on the raw quantitative data, which consisted of pixel values for each protein band quantified. Two-tailed student t-Test with equal variance was used to estimate the probability that differences in the levels of each protein, between genotypes, resulted from random chance (p values for each comparison are noted on the figure). Because the levels of each protein were measured as lower in mice lacking one RAP allele, we chose to graph the data by setting the mean value for each protein in the [APPswe/PS1dE9](+/−)/RAP(+/+) mice to 100, designated controls, and then graphing the values of mice lacking one RAP allele as a percent of the controls. The data represent measures from 6 [APPswe/PS1dE9](+/−)/RAP(+/+) mice compared to 7 [APPswe/PS1dE9] (+/−)/RAP(+/−) mice (except for measures of LR11 n = 6 as one lane was unmeasurable). The two mice without APPswe/PS1dE9 transgenes were not included in measurements so that the only difference between the two groups of animals analyzed was the number of functional RAP alleles.

**Figure 4:**
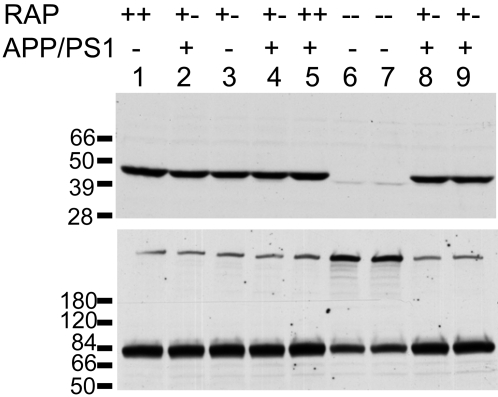
Analysis of LRP and RAP levels in transgenic animals. Five [APPswe/PS1dE9](+/−)/RAP(+/−) mice (lanes 2, 4, 8, 9), one [APPswe/PS1dE9](−)/RAP(+/+) mouse (lane 1), one [APPswe/PS1dE9](−)/RAP(+/−) mouse (lane 3), one [APPswe/PS1dE9](+/−)RAP(+/+) mouse (lane 5) and two [APPswe/PS1dE9](−)/RAP(−/−) mice (lanes 6, 7) were used in this experiment. 100 µg total protein was loaded per lane. Upper panel - Immunoblot of tissue extract probed with antibody 4109 to RAP (1∶1000). Lower panel - Immunoblot of tissue extract probed with antibody 377 to LRP (1∶1000). The upper band (∼600 kDa) is the full length LRP, the lower band is an 85 kDa fragment of mature, endoproteolytically cleaved, LRP.

**Figure 5:**
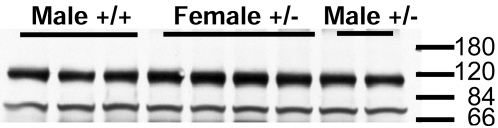
Analysis of APP levels in transgenic mice. The tissue samples used in this experiment were from the same preparation as those in Figure 4. Immunoblots were probed with monoclonal antibody, 6E10, which is specific for human Aβ 1–17 amino acids contained within the transgene product. Each lane contains 50 µg total protein. Lanes 1–3 were male APPswe/PS1dE9(+/−) mice with RAP(+/+), Lane 4–7 were female APPswe/PS1dE9(+/−) mice with RAP(+/−), Lane 8 and 9 were male APPswe /PS1dE9 mice with RAP(+/−). No obvious difference of the levels of full-length APPswe protein was noted.

Immunoblot analysis of tissue homogenates from the forebrain of mice from the different genotypes was used to analyze the levels of RAP, LRP, and SorLA/LR11 (Figs. 3A and B). As expected, mice harboring one disabled RAP allele produced about 50% less RAP protein (Figs. 3A and B). By contrast, the levels of LRP and SorLA/LR11 did not show the same level of reduction (Figs. 3A and B). On average, the levels of LRP were reduced 20%, but variability between animals reduced statistical significance to only a trend (p<0.20). The levels of SorLA/LR11, however, were less variable and were measured as reduced on average by 15% (p<0.05). Thus, the levels of both of these RAP-dependent receptors were modestly reduced in mice partially deficient in RAP. As previously reported in the literature [28], the brains of mice lacking the expression of RAP showed marked reductions in the levels of mature endoproteolytically processed LRP (85 KDa) with higher levels of immature LRP (Fig. 4, lanes 6 and 7). Surprisingly, the co-expression of PS1dE9 with partial reductions in RAP did not lead to more robust reductions in the levels of mature LRP (Fig. 4, lanes 2, 4, 5, 8, and 9 compared to lanes 1 and 3). Immunoblot analyses were also used to examine whether reducing RAP levels had an effect on the levels of full-length APPswe protein. Immunoblots with antibody 6E10, which specifically recognizes the human Aβ domain of the transgene product, revealed similar amounts of full length APP in the brains of mice with the two RAP genotypes (+/+ and +/−) (Fig. 5). Together, we find that the partial loss of RAP modestly lowers the levels of mature LRP and SorLA/LR11 in the brains of mice that co-express APPswe/PS1dE9.

## Discussion

The goal of the present investigation was to examine the roles of RAP, and indirectly of LRP, on amyloidogenesis by reducing the levels of RAP. As a result of unexpected lethality in mice that expressed APPswe/PS1dE9 in a RAP null background, we focused our analysis on mice with only partial reductions of RAP, finding that partial reduction increased the rate of Aβ deposition by 1.5 to 2-fold. By our estimates, 50% reductions in the level of RAP cause 15–20% reductions in the levels of mature LRP and SorLA/LR11. We expected that mice co-expressing mutant PS1dE9 with partial reductions of RAP would exhibit larger affects on LRP maturation because mice that are wild-type for RAP and transgenic for PS1-M146L or L286V show 40% reductions in the levels of mature LRP [27]. However, we did not observe an obvious effect of PS1dE9 expression on LRP processing. Thus, the strongest correlate to increased Aβ deposition in this experiment is the 50% reduction in RAP levels.

A caveat of our study is that the [APPswe/PS1dE9](+/−)/RAP+/− mice and the [APPswe/PS1dE9](+/−)/RAP(+/+) mice they were compared to were of a slightly different genetic background. The RAP knockout mice were congenic on the C57BL/6J background while the parental APPswe/PS1dE9 mice were hybrids of C57BL/6J and C3H/HeJ (maintained by crosses of transgenic males to F1 hybrids of the 2 strains purchased from Jackson Laboratory). In prior studies of mice that harbor the APPswe and mutant PS1 transgene constructs utilized here, we have noted that transfer of the transgene from the hybrid background to C57BL/6J mice modestly delays the onset of amyloid deposition to lower amyloid loads at younger ages [34]. Therefore, we have confidence that the increased amyloid burden seen in first generation offspring of APPswe/PS1dE9 mice×RAP knockout mice is due to the partial loss of RAP and not to effects of the other genetic factors in the backgrounds of these mouse strains. Indeed, the influence of strain background may have attenuated the magnitude of the change in amyloid burden by the partial reduction in RAP.

Whether modest reductions in LRP or SorLA/LR11 account for the increased amyloid burden we have observed is unclear. In previous studies, complete deficiency in SorLA/LR11 was shown to cause a 30% increase in the secretion of mouse Aβ40 and 42 [35]. More substantial reductions in SorLA/LR11 levels (80%) in the frontal cortex of AD patients, without changes in LRP levels, has been found to correlate with higher amyloid burden [35]. In the study by Van Uden and colleagues of PDGF-APP_Ind_ mice mated to RAP KO mice, increases in amyloid burden were correlated with 80% reductions in the levels of mature LRP [27]. Collectively, these studies suggest that reductions in LRP and/or SorLA/LR11 that are of a significantly greater magnitude than found here produce significant changes in amyloidogenesis. We conclude that either RAP possesses an activity that influences amyloidogenesis in the absence of profound effects on LRP and SorLA/LR11 maturation, or relatively modest reductions in the levels of these proteins (separately or in combination) are sufficient to cause significant changes in the rate of amyloid deposition in the APPswe/PS1dE9 model.

The genetic association of LRP and SorLA/LR11, and their ligands, APOE, α2M, and APP, to AD indicates that these membrane receptor proteins could play important roles in the pathogenesis of AD. Previous studies provide evidence that LRP has opposing effects on APP processing and Aβ metabolism. LRP can promote Aβ production by altering the processing of APP through interactions with the Kunitz protease inhibitor (KPI) domain (APP751 or APP770) [36]. Although the APP695 isoform, which lacks a KPI domain, can weakly bind to LRP through cytoplasmic adaptor proteins, such as FE65 [37], it is not known whether the processing of APP695 can be influenced by LRP. LRP also functions in the catabolism of Aβ peptides and it is possible that modest reductions in LRP levels are sufficient to diminish the rate of Aβ clearance, which involves the binding of Aβ to the LRP ligands ApoE or α2M [19], [21]–[23], [38], [39]. SorLA/LR11 has been shown to promote the trafficking of APP to discrete intracellular compartments that result in decreased Aβ secretion [35]. Thus, reduced levels of SorLA/LR11 could increase Aβ production. Collectively, these studies suggest very complicated inter-connected pathways by which these proteins could influence APP processing and Aβ metabolism. It is possible that the combined effects of modest reductions in both LRP and SorLA/LR11 could, by different mechanisms, alter the balance of Aβ production and clearance to increase the rate of amyloid deposition. However, because the effects of partial RAP deficiency in the levels of these two proteins is so modest, it is possible that RAP may have the capacity to modulate amyloid deposition by other, yet to be defined, pathways.

As outlined in the Introduction, our work replicates and extends a previous study by van Uden and colleagues [28]. Despite the potential caveats to the van Uden study that are described in the Introduction, we seem to produce a similar result, which is increased amyloid burden when RAP levels are decreased. In our case, only partial reduction in RAP levels was sufficient to increase amyloid deposition. Although we have similar outcomes to the van Uden study, the mechanisms by which these effects occur could be different. The J9 mice used by van Uden were PDGF-hAPP_Swe/Ind_ mice, which created by Mucke and colleagues [40]. The J9 model utilizes a mini-APP gene that can produce all three isoforms of APP: APP695, APP751 and APP770 [41]. Thus, based on the discussion above, one prediction might have been for deficiency in RAP to lead to diminished amyloid deposition because the absence of LRP would reduce Aβ production from APP751 and 770 splice variants. However, since amyloid burden increased in RAP deficiency, it is possible that the loss of LRP slowed the clearance of Aβ, resulting in increased amyloid deposition. It is also possible that lowering the levels of SorLA/LR11 by deleting RAP may have contributed to the increase in Aβ deposition.

In a direct test of the role of LRP in amyloidogenesis, transgenic mice that overexpress an LRP-minireceptor, containing the ligand-binding domain II of human LRP and the region representing the transmembrane subunit including the full cytoplasmic tail (mLRP2), were mated to PDAPP mice [24]. The authors reported 3-fold increases in LRP levels and found increased soluble Aβ and enhanced memory impairment in PDAPP mice, but did not find evidence of a change in amyloid loads in mice harboring both transgenes as compared to mice harboring only the PDAPP transgene. If LRP directly affects amyloidogenesis by mediating clearance of Aβ peptides, then one would have predicted that the above study would have produced mice with lowered amyloid burden. However, the transgene product was not full-length LRP and the transgene was not expressed by its endogenous promoter. Additional study is clearly required to clarify the role LRP in APP and Aβ metabolism.

### Mutant PS1 and LRP maturation

It has been reported that the levels of mature, endoproteolytically cleaved, LRP in the murine nervous system are reduced when AD associated mutants of PS1 (M146L or L286V, expressed via the hamster prion protein gene promoter) are present [27]. Our expectation was that PS1dE9 (expressed via the mouse prion protein gene promoter), which has a strong effect on APP processing [33], [42], [43], would also affect LRP maturation. However, our data do not provide evidence that this is the case. Moreover, partial reduction of RAP with co-expression of PS1dE9 also has little impact on the levels of mature LRP. Thus, either the PS1dE9 variant of PS1 does not have the same activity towards LRP maturation as possessed by the M146L or L286V variants, or some subtle difference in the expression pattern of the PS1 transgenes in the different mouse models modulates the effects.

### Lethality in RAP(−/−) mice that co-express APPswe/PS1dE9

The cause of lethality in RAP(−/−) mice that harbor the APPswe/PS1dE9 transgene is not clear. The overall number of RAP(−/−) mice, regardless of the presence of the APPswe/PS1dE9 transgene, that were identified in the genotyping of weaning age mice was lower than expected (Table 1). Initially, we suspected that the poor survival of [APPswe/PS1dE9](+/−)/RAP(−/−) mice was due to a combined effect of RAP deficiency and mutant PS1expression on LRP levels such that LRP levels were lowered to a level below the threshold for survival. Homozygous deletion of LRP is embryonic lethal [44]. However, as noted above, LRP maturation in PS1dE9 mice was not affected as significantly as reported in mice expressing other PS1 variants. At present the basis for poor survival is unknown but it appears that expression of PS1dE9 is implicated because others have not noted that expression of mutant APP in a RAP null background decreases survival [27].

### Conclusions

It is clear from the literature that interactions between RAP and LRP, between LRP and APP, and between LRP and APOE, α2M, and Aβ create a complicated network by which amyloidogenesis might be regulated. RAP also regulates other members of the LRP family, such as LRP1B [17], SorLA/LR11[45], LDL-R and VLDL-R [46], which adds additional complexity to the system. We find that APPswe/PS1dE9 mice with partial reduction in RAP showed significant amyloid burden increases with modest to slight reductions in the levels of mature SorLA/LR11 and LRP. Although it is possible that modest reductions in SorLA/LR11 and LRP act in concert to alter the processing or clearance of Aβ, it is also possible that RAP has more direct effects on APP processing or the clearance of Aβ peptides. Overall, RAP could be viewed as either directly or indirectly influencing multiple processes that modulate Alzheimer-type amyloidogenesis.

## References

[1] Vetrivel KS, Thinakaran G (2006). Amyloidogenic processing of beta-amyloid precursor protein in intracellular compartments.. Neurology.

[2] Corder EH, Saunders AM, Strittmatter WJ, Schmechel DE, Gaskell PC (1993). Gene dose of apolipoprotein E type 4 allele and the risk of Alzheimer's disease in late onset families.. Science.

[3] Kang DE, Saitoh T, Chen X, Xia Y, Masliah E (1997). Genetic association of the low-density lipoprotein receptor-related protein gene (LRP), an apolipoprotein E receptor, with late-onset Alzheimer's disease.. Neurology.

[4] Rogaeva E, Meng Y, Lee JH, Gu Y, Kawarai T (2007). The neuronal sortilin-related receptor SORL1 is genetically associated with Alzheimer disease.. Nat Genet.

[5] Clatworthy AE, Gomez-Isla T, Rebeck GW, Wallace RB, Hyman BT (1997). Lack of association of a polymorphism in the low-density lipoprotein receptor-related protein gene with Alzheimer disease.. Arch Neurol.

[6] Fallin D, Kundtz A, Town T, Gauntlett AC, Duara R (1997). No association between the low density lipoprotein receptor-related protein (LRP) gene and late-onset Alzheimer's disease in a community-based sample.. Neurosci Lett.

[7] Wavrant-DeVrieze F, Perez-Tur J, Lambert JC, Frigard B, Pasquier F (1997). Association between the low density lipoprotein receptor-related protein (LRP) and Alzheimer's disease.. Neurosci Lett.

[8] Scott WK, Yamaoka LH, Bass MP, Gaskell PC, Conneally PM (1998). No genetic association between the LRP receptor and sporadic or late-onset familial Alzheimer disease.. Neurogenetics.

[9] Willnow TE (1999). The low-density lipoprotein receptor gene family: multiple roles in lipid metabolism.. J Mol Med.

[10] Herz J, Kowal RC, Goldstein JL, Brown MS (1990). Proteolytic processing of the 600 kd low density lipoprotein receptor-related protein (LRP) occurs in a trans-Golgi compartment.. Embo J.

[11] Bu G, Geuze HJ, Strous GJ, Schwartz AL (1995). 39 kDa receptor-associated protein is an ER resident protein and molecular chaperone for LDL receptor-related protein.. Embo J.

[12] Willnow TE (1998). Receptor-associated protein (RAP): a specialized chaperone for endocytic receptors.. Biol Chem.

[13] Van Uden E, Kang DE, Koo EH, Masliah E (2000). LDL receptor-related protein (LRP) in Alzheimer's disease: towards a unified theory of pathogenesis.. Microsc Res Tech.

[14] Yoon IS, Pietrzik CU, Kang DE, Koo EH (2005). Sequences from the low density lipoprotein receptor-related protein (LRP) cytoplasmic domain enhance amyloid beta protein production via the beta-secretase pathway without altering amyloid precursor protein/LRP nuclear signaling.. J Biol Chem.

[15] Ulery PG, Beers J, Mikhailenko I, Tanzi RE, Rebeck GW (2000). Modulation of beta-amyloid precursor protein processing by the low density lipoprotein receptor-related protein (LRP). Evidence that LRP contributes to the pathogenesis of Alzheimer's disease.. J Biol Chem.

[16] Kounnas MZ MR, Rebeck GW, Bush AI, Argraves WS, Tanzi RE (1995). LDL receptor-related protein, a multifunctional ApoE receptor, binds secreted beta-amyloid precursor protein and mediates its degradation.. Cell.

[17] Cam JA, Zerbinatti CV, Knisely JM, Hecimovic S, Li Y (2004). The Low Density Lipoprotein Receptor-related Protein 1B Retains beta-Amyloid Precursor Protein at the Cell Surface and Reduces Amyloid-beta Peptide Production.. J Biol Chem.

[18] Cam JA, Zerbinatti CV, Li Y, Bu G (2005). Rapid Endocytosis of the Low Density Lipoprotein Receptor-related Protein Modulates Cell Surface Distribution and Processing of the beta-Amyloid Precursor Protein.. J Biol Chem.

[19] Kang DE, Pietrzik CU, Baum L, Chevallier N, Merriam DE (2000). Modulation of amyloid beta-protein clearance and Alzheimer's disease susceptibility by the LDL receptor-related protein pathway.. J Clin Invest.

[20] Causevic M, Ramoz N, Haroutunian V, Davis KL, Buxbaum JD (2003). Lack of association between the levels of the low-density lipoprotein receptor-related protein (LRP) and either Alzheimer dementia or LRP exon 3 genotype.. J Neuropathol Exp Neurol.

[21] Qiu Z, Strickland DK, Hyman BT, Rebeck GW (1999). Alpha2-macroglobulin enhances the clearance of endogenous soluble beta-amyloid peptide via low-density lipoprotein receptor-related protein in cortical neurons.. J Neurochem.

[22] Shibata M, Yamada S, Kumar SR, Calero M, Bading J (2000). Clearance of Alzheimer's amyloid-ss(1-40) peptide from brain by LDL receptor-related protein-1 at the blood-brain barrier.. J Clin Invest.

[23] Narita M, Holtzman DM, Schwartz AL, Bu G (1997). Alpha2-macroglobulin complexes with and mediates the endocytosis of beta-amyloid peptide via cell surface low-density lipoprotein receptor-related protein.. J Neurochem.

[24] Zerbinatti CV, Wozniak DF, Cirrito J, Cam JA, Osaka H (2004). Increased soluble amyloid-beta peptide and memory deficits in amyloid model mice overexpressing the low-density lipoprotein receptor-related protein.. Proc Natl Acad Sci U S A.

[25] Herz J, Clouthier DE, Hammer RE (1992). LDL receptor-related protein internalizes and degrdes uPA-PAI-1 complexes and is essential for embryo implantation.. Cell.

[26] Willnow TE, Armstrong SA, Hammer RE, Herz J (1995). Functional expression of low density lipoprotein receptor-related protein is controlled by receptor-associated protein in vivo.. Proc Natl Acad Sci U S A.

[27] Van Uden E, Carlson G, St George-Hyslop P, Westaway D, Orlando R (1999). Aberrant presenilin-1 expression downregulates LDL receptor-related protein (LRP): is LRP central to Alzheimer's disease pathogenesis?. Mol Cell Neurosci.

[28] Van Uden E, Mallory M, Veinbergs I, Alford M, Rockenstein E (2002). Increased extracellular amyloid deposition and neurodegeneration in human amyloid precursor protein transgenic mice deficient in receptor-associated protein.. J Neurosci.

[29] Jankowsky JL, Slunt HH, Ratovitski T, Jenkins NA, Copeland NG (2001). Co-expression of multiple transgenes in mouse CNS: a comparison of strategies.. BiomolEng.

[30] Yamamoto T, Hirano A (1986). A comparative study of modified Bielschowsky, Bodian and thioflavin S stains on Alzheimer's neurofibrillary tangles.. Neuropathol Appl Neurobiol.

[31] Jankowsky JL, Slunt HH, Gonzales V, Savonenko AV, Wen JC (2005). Persistent amyloidosis following suppression of Abeta production in a transgenic model of Alzheimer disease.. PLoS Med.

[32] Xu G, Gonzales V, Borchelt DR (2002). Rapid detection of protein aggregates in the brains of Alzheimer patients and transgenic mouse models of amyloidosis.. Alzheimer Dis Assoc Disord.

[33] Lesuisse C, Xu G, Anderson J, Wong M, Jankowsky J (2001). Hyper-expression of human apolipoprotein E4 in astroglia and neurons does not enhance amyloid deposition in transgenic mice.. Hum Mol Genet.

[34] Savonenko AV, Xu GM, Price DL, Borchelt DR, Markowska AL (2003). Normal cognitive behavior in two distinct congenic lines of transgenic mice hyperexpressing mutant APP SWE.. Neurobiol Dis.

[35] Andersen OM, Reiche J, Schmidt V, Gotthardt M, Spoelgen R (2005). Neuronal sorting protein-related receptor sorLA/LR11 regulates processing of the amyloid precursor protein.. Proc Natl Acad Sci U S A.

[36] Rebeck GW, Moir RD, Mui S, Strickland DK, Tanzi RE (2001). Association of membrane-bound amyloid precursor protein APP with the apolipoprotein E receptor LRP.. Brain Res Mol Brain Res.

[37] Pietrzik CU, Yoon I-S, Jaeger S, Busse T, Weggen S (2004). FE65 Constitutes the Functional Link between the Low-Density Lipoprotein Receptor-Related Protein and the Amyloid Precursor Protein.. J Neurosci.

[38] Herz J, Strickland DK (2001). LRP: a multifunctional scavenger and signaling receptor.. J Clin Invest.

[39] Deane R, Wu Z, Zlokovic BV (2004). RAGE (yin) versus LRP (yang) balance regulates alzheimer amyloid beta-peptide clearance through transport across the blood-brain barrier.. Stroke.

[40] Mucke L, Masliah E, Yu GQ, Mallory M, Rockenstein EM (2000). High-level neuronal expression of abeta 1-42 in wild-type human amyloid protein precursor transgenic mice: synaptotoxicity without plaque formation.. J Neurosci.

[41] Games D, Adams D, Alessandrini R, Barbour R, Berthelette P (1995). Alzheimer-type neuropathology in transgenic mice overexpressing V717F beta-amyloid precursor protein.. Nature.

[42] Jankowsky JL, Fadale DJ, Anderson J, Xu GM, Gonzales V (2004). Mutant presenilins specifically elevate the levels of the 42 residue beta-amyloid peptide in vivo: evidence for augmentation of a 42-specific gamma secretase.. Hum Mol Genet.

[43] Thinakaran G, Borchelt DR, Lee MK, Slunt HH, Spitzer L (1996). Endoproteolysis of presenilin 1 and accumulation of processed derivatives in vivo.. Neuron.

[44] Herz J, Clouthier DE, Hammer RE (1992). LDL receptor-related protein internalizes and degrades uPA-PAI-1 complexes and is essential for embryo implantation.. Cell.

[45] Jacobsen L, Madsen P, Moestrup SK, Lund AH, Tommerup N (1996). Molecular characterization of a novel human hybrid-type receptor that binds the alpha2-macroglobulin receptor-associated protein.. J Biol Chem.

[46] Veinbergs I, Van Uden E, Mallory M, Alford M, McGiffert C (2001). Role of apolipoprotein E receptors in regulating the differential in vivo neurotrophic effects of apolipoprotein E.. Exp Neurol.

